
JOURNAL INFORMATION
==============================
NLM Title Abbreviation: ScientificWorldJournal
Journal ID: ScientificWorldJournal
NLM Journal ID: 101131163
Journal ID: TSWJ

The Scientific World Journal

ISSN: 2356-6140
EISSN: 1537-744X
Publisher: Wiley

ARTICLE INFORMATION
==============================
PMCID: PMC6009327
PMID: 29973842
DOI: 10.1100/tsw.2002.65
Article ID: 478639
Article version: 1
Subjects: Short Report

Expression of Heme Oxygenase-1 Mediated by A Protein Transduction Domain Protects Insulin Producing Cells from Cytokine- Induced Cytotoxicity

Ribeiro Melina *
Klein Dagmar
Pileggi Antonello
Molano R. Damaris
Fraker CHristopher
Ricordi Camillo
Inverardi Luca
Diabetes Research Institute, University of Miami School of Medicine, Miami, Fl 33136, USA
*Melina Ribeiro: rpastori@med.miami.edu
Publication date: 2002
Electronic publication date: 2002 May 22
Volume: 2
First page: 138
Last page: 139
Copyright: Copyright © 2002 Melina Ribeiro et al.
Copyright year: 2002
License: This is an open access article distributed under the Creative Commons Attribution License, which permits unrestricted use, distribution, and reproduction in any medium, provided the original work is properly cited.
License URL: https://creativecommons.org/licenses/by/3.0/

==============================
Advances in Gene Technology: The Genome and Beyond –
Structural Biology for Medicine (Proceedings of the 2002
Miami *Nature Biotechnology* Winter Symposium)
*TheScientificWorld* 2002, 2(S2), 138–139
ISSN 1532-2246; DOI 10.1100/tsw.2002.65

### EXPRESSION OF HEME OXYGENASE-1 MEDIATED BY A PROTEIN TRANSDUCTION DOMAIN PROTECTS INSULIN PRODUCING CELLS FROM CYTOKINE- INDUCED CYTOTOXICITY

Melina Ribeiro, Dagmar Klein, Antonello Pileggi, R. Damaris Molano, Christopher Fraker, Camillo Ricordi, Luca Inverardi, and Ricardo L. Pastori
Diabetes Research Institute, University of Miami School of Medicine, Miami, Fl 33136
*rpastori@med.miami.edu

**INTRODUCTION.** Microsomal Heme Oxygenase-1 (HO-1) has been identified as a ubiquitous stress protein induced in many cell types by various stimulants such as hemolysis, inflammatory agents, oxidative stress, heat shock, and growth factors[1]. HO-1 is the enzyme that controls the degradation pathway of heme by catalyzing its oxidation into biliverdin, carbon monoxide, and iron. Although the role of HO-1 induction in oxidative stress is not completely understood, it has been shown that induction of HO-1 expression results in protection from cytokine-induced apoptosis and oxidative stress in *in vitro* cell culture and in various animal models, including pancreatic islet transplantation[2]. Thus, expression of HO-1 through gene therapy protocols might prove useful to reduce the deleterious effects of oxidative stress in islet isolation as well as to prevent damage in the peri-transplant period. However, viral vectors or other transfection methods available for transduction of genes into islets have limited efficacy and the presence of viral antigens (especially adenovirus) may potentially induce immunological responses against the transfected islets. Moreover, the long-term effects of genetic manipulations of islets, in particular those affecting apoptosis, may have undesirable long-term effects such as impaired mitochondrial signals regulating insulin secretion. Proteins can be directly transferred to cells when they are linked to protein transduction domains (PTDs), small peptide domains that can freely cross cell membranes[3]. In particular, proteins fused to an 11-amino acid protein transduction domain (PTD) from the human immunodeficiency virus transactivator of transcription (TAT) protein, readily transduce many cell types including pancreatic islets[4]. In this study we have characterized a functional HO-1 protein fused to a PTD

**METHODS.**

**Cloning and Related Techniques**. The recombinant TAT antiapoptotic fusion proteins were generated by subcloning the coding region of the murine HO-1 gene in frame with the TAT leader peptide (amino acids 47-58 YGRKKRRQRRR) in a bacterial expression generously provided by Steven Dowdy from Washington University School of Medicine, St Louis. A 6xHis-affinity tag allowed the purification of the fusion protein through affinity chromatography.

**RESULTS**

- • The TAT-PTD/HO-1 fusion protein transduced insulin-producing cells in a time- and dose-dependent way, while retaining its biological function.
- • Transduction of TAT-PTD/HO-1 to insulin-producing cells protects them from cytokines-mediated cytotoxicity.
- • The TAT-PTD/HO-1fusion protein is also capable to transduce pancreatic islets in culture, dramatically improving their viability.

**DISCUSSION.** From our results we conclude that heme oxygenase-1 fused with the TAT/PTD can be transduced into insulin producing cells as an enzymatically active protein, exhibiting cytoprotective effects.

**REFERENCES**

1. Petrache, I., Otterbein, L.E., Alam, J., Wiegand, G.W., and Choi, A.M. (2000) Am. J. Physiol. Lung. Cell Mol. Physiol. 278, L312–L319.
2. Pileggi, A., Molano, R.D., Berney, T., Cattan, P., Vizzardelli, C., Oliver, R., Fraker, C., Ricordi, C., Pastori, R.L., and Inverardi, L. (2001) Diabetes, 50, 1983–1991.
3. Schwarze, S.R., Ho, A., Vocero-Akbani, A., and Dowdy, S.F. (1999) Science 285, 1569–1572.
4. Embury, J., Klein, D., Pileggi, A., Ribeiro, M., Jayaraman, S., Molano, R.D., Fraker, C., Kenyon, N., Ricordi, C., Inverardi, L., and Pastori, R.L. (2001) Diabetes 50, 1706–1713.
